# Expectant management of pneumothorax in intubated COVID-19 positive patients: a case series

**DOI:** 10.1186/s13019-020-01297-7

**Published:** 2020-09-21

**Authors:** Colby Elder, Sheina Bawa, Douglas Anderson, Stephen Atkinson, Joshua Etzel, Troy Moritz

**Affiliations:** 1 UPMC Pinnacle Harrisburg, 205 S. Front St. Brady Hall 9th Floor, Room 96, Harrisburg, PA 17104 USA; 2 UPMC Pinnacle Community Osteopathic, 4300 Londonderry Rd, Harrisburg, PA 17109 USA

**Keywords:** COVID-19, Pneumothorax, Case report

## Abstract

**Background:**

There is an increasing amount of literature describing the pathogenesis of coronavirus disease 2019 (COVID-19) pneumonia and its associated complications. Historically, a small pneumothorax has been shown to be successfully treated without chest tube insertion, but this management has yet to be proven in COVID-19 pneumonia patients. In addition, pneumothorax in an intubated patient with high positive end-expiratory pressure (PEEP) provides additional uncertainty with pursuing non-operative management.

**Case presentation:**

In this series we report four cases of patients with respiratory distress who tested positive for COVID-19 via nasopharyngeal swab and developed ventilator-induced pneumothoraces which were successfully managed with observation alone.

**Conclusions:**

Management of patients with COVID-19 pneumonia on positive pressure ventilation who develop small stable pneumothoraces can be safely observed without chest tube insertion.

## Introduction

COVID-19 pneumonia may cause cystic features of lung parenchyma which can resolve or progress to larger blebs [1, 2]. This can place patients at risk for rupture resulting in mediastinal and subcutaneous emphysema or secondary spontaneous pneumothorax. Many are intubated and placed on low tidal volume and high PEEP ventilation therapy which further increases concern for rupture. For critically ill patients on positive pressure ventilation, although controversial, it is currently recommended to place a tube thoracostomy when a pneumothorax is observed [3]. Due to limited knowledge of lung histopathology with COVID-19, it is unknown how well the diseased lung tissue will spontaneously heal and re-expand without intervention. For moderate to large pneumothorax and prolonged air leak, there have been reports of successful treatment with video-assisted thoracoscopic surgery and wedge resection [4]. There have also been explanations on how to contain viral dissemination by using bespoke viral filtration systems to limit contamination [5]. However, for a small pneumothorax in a stable patient, chest tube placement may be second line to watchful waiting. We present four cases of pneumothorax in COVID-19 positive patients who were managed without chest tube placement despite being on positive pressure ventilation.

## Cases

### Case 1

**Fig. 1:**
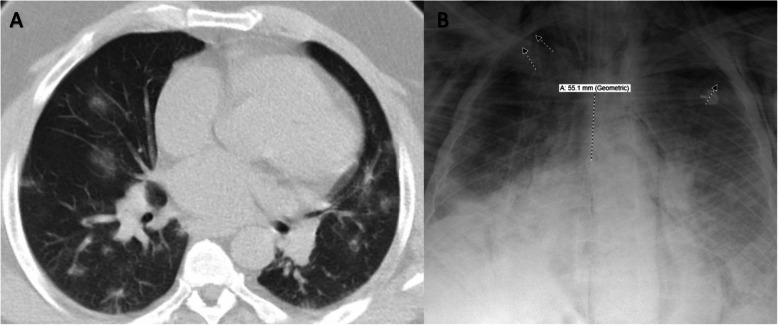
**a** Chest CT showing bilateral ground glass opacities. **b** Chest X-ray showing subcutaneous emphysema and bilateral tiny apical pneumothoraces (arrows show pleural lines)

A 48-year-old male with hypertension and hyperlipidemia without a history of cigarette smoking presented to the emergency (ED) with 1 week of a worsening dry cough associated with chest pain, headaches, myalgia, shortness of breath, and subjective fevers. A chest computed tomography (CT) showed bilateral ground glass opacities (Fig. 1a). Laboratory workup showed white blood cell count of 9000 /μL, c-reactive protein 8.66, creatinine 1.32 mg/dL, and procalcitonin 0.14 mg/dL. The next day he was confirmed COVID positive. He soon developed acute respiratory insufficiency, required intubation, and was transferred to the intensive care unit. Ventilatory settings were PEEP 16 cm H2O, tidal volume (VT) 6 mL/kg, and target plateau pressure 30–55 cm H2O. His FiO2 was weaned to 40% and on hospital day 4 his chest x-ray (CXR) showed extensive subcutaneous emphysema and bilateral tiny apical pneumothoraces (Fig. 1b). The PEEP was then decreased to 14. A follow up CXR showed worsening pneumomediastinum and subcutaneous emphysema, but no definite pneumothorax. The next day he required a PEEP of 16, but never redeveloped a pneumothorax for the remainder of his hospital course. He eventually received a tracheostomy and has since been weaned from the ventilator. His tracheostomy tube has been downsized and over 3 months later chest x-rays showed no pneumothorax.

### Case 2

**Fig. 2:**
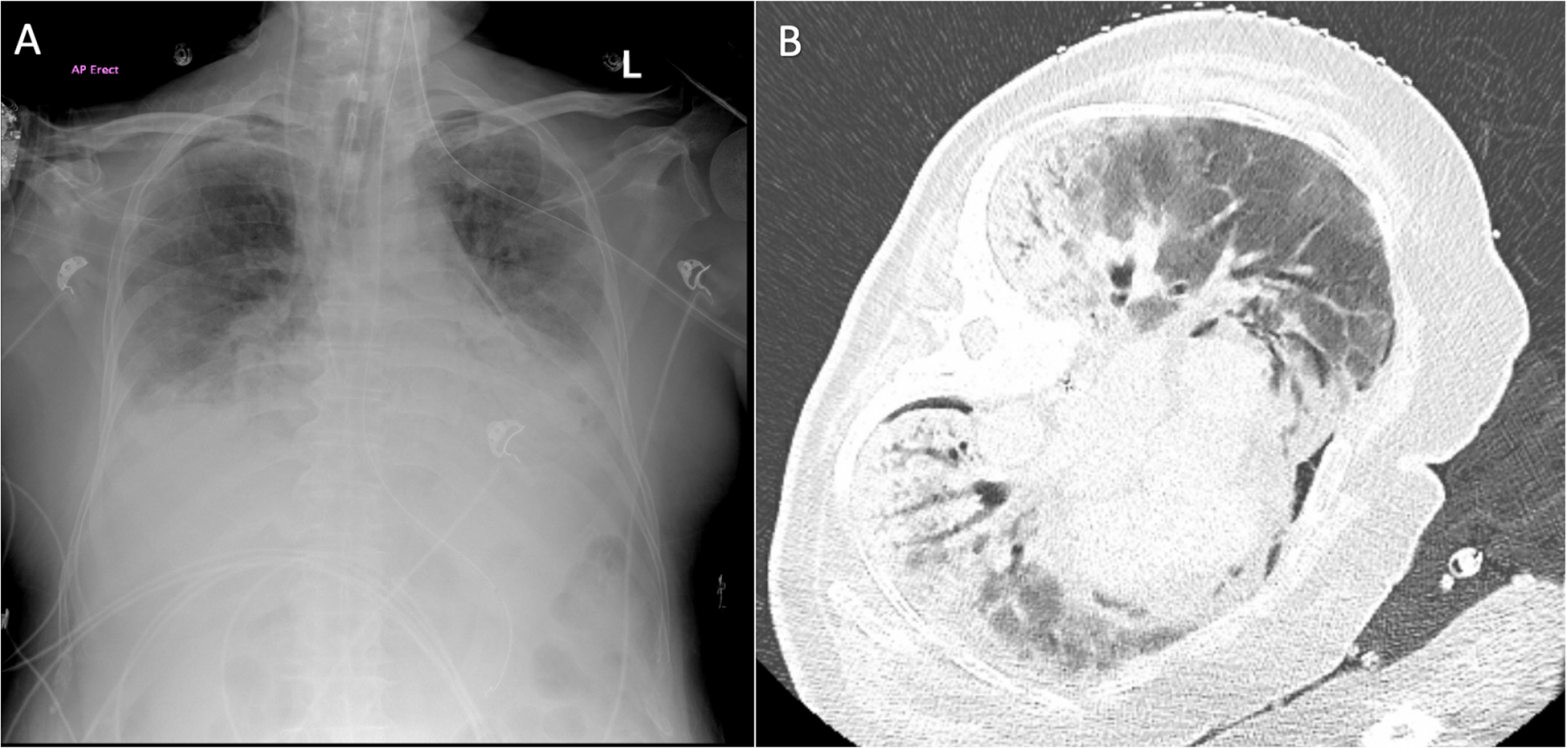
**a** Chest X-ray revealing small bilateral apical pneumothoraces. **b** Chest CT showing Trace bilateral apical pneumothoraces with extensive pneumomediastinum

A 76-year-old male presented to the ED with shortness of breath and excessive dry coughing. The patient had tested positive for COVID-19 3 days prior but was sent home to recover. Over the course of those 3 days, the patient got progressively worse, with decreased O2 saturations and increased work of breathing. He also began to have worsening cough and persistent fevers while at home. Upon return to the ED, he was severely hypoxic with increased work of breathing and required intubation within 36 h of presentation. Treatment was initiated with azithromycin, hydroxychloroquine, zinc, tocilizumab, and dexamethasone for his COVID-19 infection. His hospital course was complicated by septic shock, acute respiratory distress syndrome (ARDS), volume overload, and uncontrolled diabetes. The patient had been intubated and on ventilator support for 21 days when a chest x-ray revealed bilateral pneumothoraces, which were confirmed with a chest CT, along with pneumomediastinum (Fig. 2). The patient was transferred to a hospital where thoracic surgery was available and imaging after transfer showed the pneumothorax had slightly increased without tension physiology. The PEEP was decreased from 8 to 6 cm H20 where it remained for the duration of the hospital course. The next day follow up chest x-rays showed resolution of pneumothoraces. On hospital day 31 CXR continued to show no pneumothorax, however the patient passed away from hypoxic respiratory failure later that day.

### Case 3

**Fig. 3:**
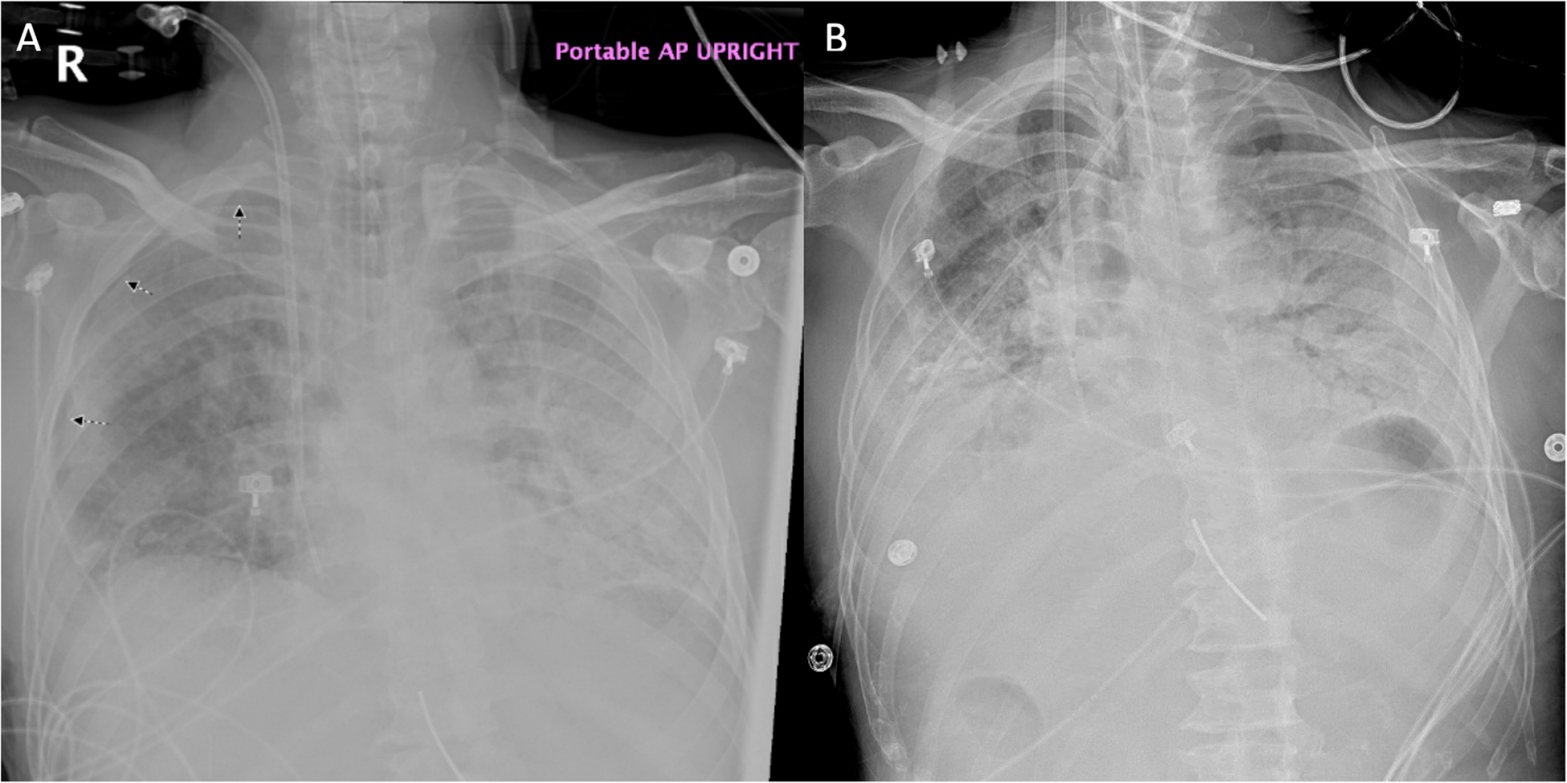
**a** Chest x-ray with small right pneumothorax (arrows). **b** Chest x-ray showing resolution of pneumothorax 1 h prior death pronouncement

This is a 68-year-old male who was exposed to a family member who was COVID-19 positive. He began complaining of fevers, cough and dizziness several days prior to presentation to the ED. He was hospitalized for workup of pre-syncope and was confirmed COVID-19 positive. His respiratory status declined and on hospital day 3 he was transferred to the ICU and intubated. Over the next 21 days the patient failed two extubation attempts secondary to hypercapnia and hypoxia. In addition, one failed extubation led to an aspiration event leading to superimposed aspiration pneumonia. The ventilator was set to pressure control of 37 cm H20 with a PEEP of 0, and never changed throughout the hospital course. A right internal jugular dialysis catheter was replaced on hospital day 28 with no pneumothorax seen on post-CXR. Three days later a small right-sided pneumothorax was noted on CXR (Fig. 3a) and thoracic surgery was consulted. Due to a stable clinical status, expectant management was pursued. A repeat x-ray 8 hours later showed the pneumothorax was stable. Three days later the patient decompensated from a respiratory standpoint and a chest x-ray that morning showed resolution of the pneumothorax (Fig. 3b). An hour later the patient died from hypoxic respiratory failure secondary to ARDS.

### Case 4

**Fig. 4:**
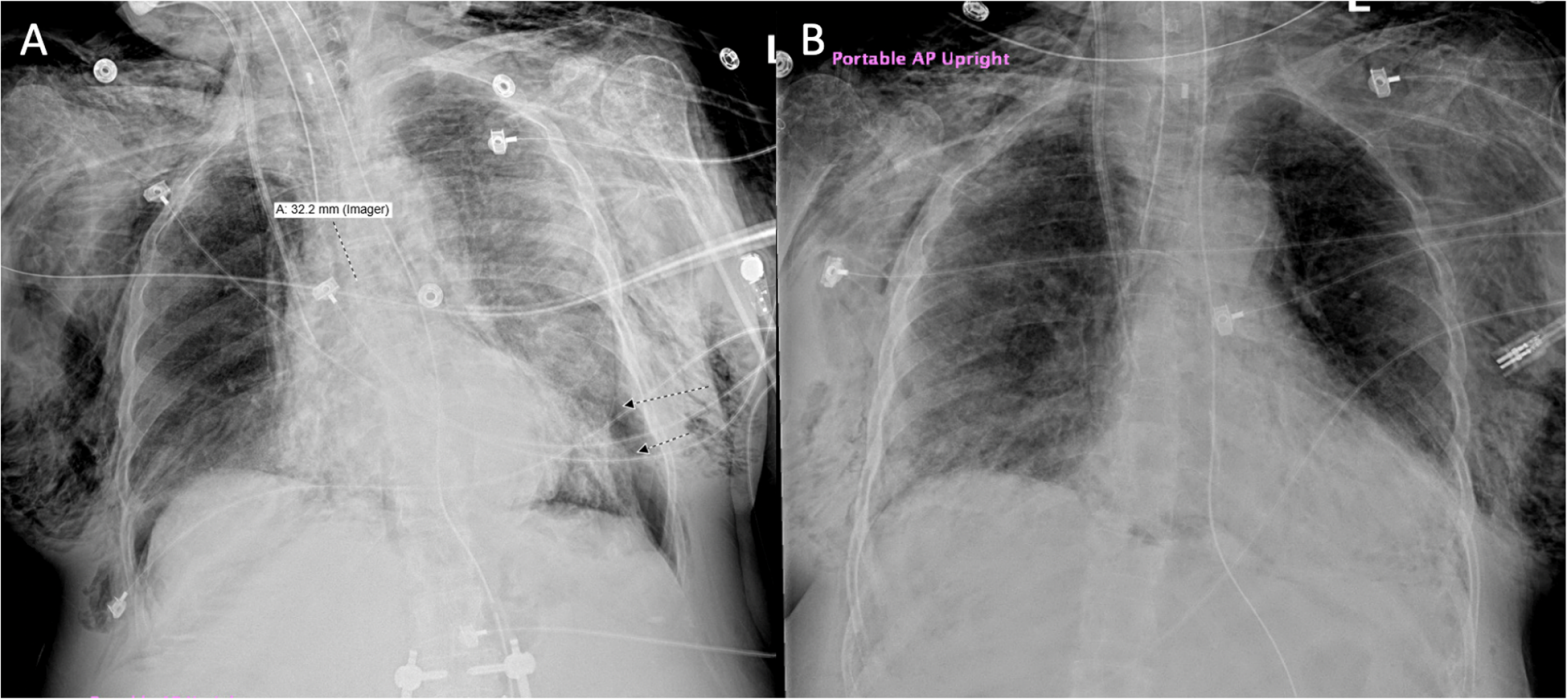
**a** Chest x-ray with small to moderate left basilar pneumothorax. **b** Follow up Chest x-ray showing tiny right apical pneumothorax

This patient is a 76-year-old female who presented to the emergency department with altered mental status, hypotension, suspected GI bleed with severe anemia, and sepsis of unknown origin. She was positive for COVID-19 pneumonia and became acutely hypoxic on hospital day 3, requiring emergent intubation. The hospital course was complicated by septic shock leading to multi organ system failure. Throughout her hospital course she continued to require vasopressor support and was ventilator dependent. Her ventilator support requirements remained stable and approximately 3 weeks into her hospital course she was found to have extensive clinical left sided subcutaneous emphysema. This prompted a CXR revealing a left small to moderate basilar pneumothorax with diffuse subcutaneous emphysema (Fig. 4a). The PEEP was decreased from 10 to 5 cm H20 in the following 24 h and expectant management was pursued. Sequential X-rays showed pneumothorax stability and 2 days later follow up chest x-ray identified an additional small right sided apical pneumothorax (Fig. 4b). Despite the decrease in vent settings, she was unable to be completely weaned from the ventilator. She had persistent tiny apical pneumothoraces until 11 days after the initial pneumothorax when CXR showed resolution of her pneumothoraces with unchanged subcutaneous emphysema. Her hospital course was complicated by renal failure and septic shock due to Staph aureus and *E. coli* pneumonia which required multiple vasopressors. She gradually became less stable and due to her prognosis, a family meeting was held to make the patient comfort measures only. She expired 21 days after initial pneumothorax seen with no evidence of redevelopment.

## Conclusions

Although there are multiple presentations, respiratory disease is the primary manifestation of a severe COVID-19 infection. For managing severe ARDS due to COVID-19, current guidelines from the United States National Institutes of Health recommend using “low tidal volume ventilation (VT 4–8 mL/kg of predicted body weight) over higher tidal volumes (VT >8 mL/kg)” and “prone ventilation for 12 to 16 hours per day over no prone ventilation” for hypoxia refractory to optimized ventilatory settings [6]. Chest CT is an instrumental part of the workup and assessment of disease severity with the most common findings being bilateral ground glass opacities. COVID-19 infection has already shown lung parenchymal changes that progressed to large bulla, which coalesce and enlarge over time leading to bullous emphysematous disease and subpleural blebs [2]. This could predispose this population to developing pneumothorax or pneumomediastinum, which have already been reported in the literature [7]. One explanation for the pneumomediastinum could be the Macklin effect, where air from ruptured alveoli dissects along bronchovascular planes and into the mediastinum [8, 9]. This is in contrast to a pneumothorax where the alveolar rupture is involved with injury to the visceral pleura resulting in air accumulation in the pleural space. A small asymptomatic primary pneumothorax can typically be managed with observation and sequential imaging to ensure stabilization or re-expansion of the lung. However, barotrauma from positive pressure ventilation is a well-known cause of iatrogenic pneumothorax and the current recommendation for critically ill patients who are intubated in the ICU is tube thoracostomy placement [3]. To the best of our knowledge, there are currently no recommendations specifically for managing a small pneumothorax in a COVID-19 positive patient. In each of these cases of pneumothorax due to barotrauma we decided to pursue non-operative management. Factors contributing to this decision were the small size of the pneumothoraces and the fact that the patient would need to be disconnected from the ventilator to allow the lung to drop to avoid parenchymal injury, which could be detrimental to an already critical patient. For the majority of our cases we decreased the PEEP as much as possible and recommend this practice to others attempting expectant management. However, further studies are needed to assess if this truly makes a difference. An additional benefit was limiting viral exposure to hospital staff. The limitations of this study is that there were only 4 cases encountered making it difficult to apply to a larger group, and that the pneumothoraces were small (< 3 cm from apex to cupola), leaving no guidance for those with moderate to large sized pneumothoraces. We have not managed any other COVID-19 patients with pneumothorax to date. While no institutional guidelines exist, we would have a lower threshold for pleural drainage in moderate to large pneumothoraces. Expectant management can be quite precarious in this population, but we believe these cases show that observation in ventilated COVID-19 positive patients with a small pneumothorax may be an appropriate option. It has been shown in post-mortem studies that COVID-19 causes diffuse alveolar damage and lymphocytic or cellular fibromyxoid exudate [10]. We hypothesize that this severe exudative process may contribute to sealing the alveolar and pleural injury, allowing for small pneumothoraces to remain stable during positive pressure ventilation. As the COVID-19 pandemic continues to unfold we will learn more about the exact pathophysiology that occurs.

## Data Availability

Not applicable.

## Abbreviations

COVID-19: Coronavirus disease 2019
PEEP: Positive end expiratory pressure
ARDS: Acute respiratory distress syndrome
ED: Emergency department
CT: Computed tomography
VT: Tidal volume
CXR: Chest x-ray
